# An event-coding account of attitudes

**DOI:** 10.3758/s13423-021-01969-y

**Published:** 2021-07-08

**Authors:** Bernhard Hommel, Niek Stevenson

**Affiliations:** 1 Institute for Psychological Research and Leiden Institute for Brain and Cognition, Leiden University, Leiden, The Netherlands; 2 Cognitive Neurophysiology, Department of Child and Adolescent Psychiatry, Faculty of Medicine, TU Dresden, Schubertstraße 42, 01307 Dresden, Germany; 3 Department of Psychology, Shandong Normal University, Jinan, China

**Keywords:** Attitudes, Social cognition, Opinion, Theory of event coding

## Abstract

Attitudes (or opinions, preferences, biases, stereotypes) can be considered bindings of the perceptual features of the attitudes’ object to affective codes with positive or negative connotations, which effectively renders them “event files” in terms of the Theory of Event Coding. We tested a particularly interesting implication of this theoretical account: that affective codes might “migrate” from one event file to another (i.e., effectively function as a component of one while actually being part of another), if the two files overlap in terms of other features. We tested this feature-migration hypothesis by having participants categorize pictures of fictitious outer space characters as members of two fictitious races by pressing a left or right key, and to categorize positive and negative pictures of the International Affective Picture System (IAPS) as positive and negative by using the same two keys. When the outer space characters were later rated for likability, members of the race that was categorized by means of the same key as positive IAPS pictures were liked significantly more than members of the race that was categorized with the same key as negative IAPS pictures – suggesting that affective feature codes from the event files for the IAPS pictures effectively acted as an ingredient of event files for the outer space characters that shared the same key. These findings were fully replicated in a second experiment in which the two races were replaced by two unfamiliar fonts. These outcomes are consistent with the claim that attitudes, opinions, and preferences are represented in terms of event files and created by feature binding.

## Introduction

Even though psychology has a long tradition of treating social and non-social cognition separately in theorizing and empirical research, there is increasing interest in possible commonalities and overlap between these fields. A strong boost in shared interests can be attributed to the social-cognition approach emerging in the early 1980s, which claimed that much can be learned from studying the cognitive processes underlying social behavior (Hamilton & Carlston, 2013). This has become the dominant approach in modern social psychology, but the theoretical profit has been meager: there is no systematic theoretical framework, apart from a few disconnected assumptions (e.g., that people are cognitive misers, and that controlled processing competes with automatic processes: Fiske & Taylor, 1991), and no systematic theory building that is guiding, and that in turn is informed by, systematic experimenting. This arguably accounts for substantial portions of the current “replication crisis” that has hit the field of social cognition particularly hard (e.g., Cairo et al., 2020). Kim and Hommel (2019) have suggested reviving the original ambition of the social-cognition approach to make use of both cognitive methods and cognitive theorizing (see also Amodio, 2019), and argued that the Theory of Event Coding (TEC: Hommel, Müsseler, Aschersleben, & Prinz, 2001) might be particularly suitable as a framework and theoretical toolbox for systematically developing cognitive theories of social phenomena. First attempts have successfully used TEC to account for some aspects of social behavior, like conformity (Kim & Hommel, 2015), trust (Hommel & Colzato, 2015), and self-other integration (Colzato et al., 2013), and the aim of the present study was to see whether TEC might also help to understand how human attitudes, opinions, and preferences are acquired and cognitively represented.

Knowing and understanding people’s attitudes, opinions, and preferences is the key to success for businesspeople and marketing engineers, but also for political leaders in democratic societies. And yet, while science has fully acknowledged the great impact and importance of these concepts on both individual behavior and societal interactions, we know very little about the mechanisms underlying their formation, maintenance, and use. These three, and other related concepts (including bias, prejudice, and stereotype; all of which we collectively refer to as “attitudes” in the following), are commonly not well defined and if they are, their definitions overlap substantially (e.g., see Fiske & Taylor, 1991). Most definitions agree on the assumption that representations of particular events, be it objects, persons, or behaviors, become bound to representations with positive and negative connotations (e.g., particular feelings, moods, or judgments). TEC assumes that people store events by binding perceptual, affective, contextual, and action-related features into event files (Hommel, 2004, 2019), suggesting that event files can be considered suitable representations of attitudes – in addition to their other functions as perceptual representations and action plans (Hommel, 2009). More specifically, attitudes can be considered to be represented by event files that bind codes of the features characterizing the state of affairs the attitude refers to (the object of the attitude) with representations of the affective state the person has experienced in the context of this state of affairs. For instance, a cherry connoisseur is likely to possess event files that bind the perceptual features of cherries, such as their round shape, their red color, and their sweet taste, with a somatic/affective marker (Damasio, Tranel, & Damasio, 1991) of the positive feeling that the person associates with being exposed to cherries.

Considering that attitudes are represented by event files offers various new predictions and experimental investigations thereof. The prediction that motivated the present study emerged from the recent TEC-inspired application of the feature-migration concept developed in studies of visual attention (Treisman & Gelade, 1980; Treisman & Schmidt, 1982) to social perception by Ma, Sellaro, Lippelt, and Hommel (2016). Treisman and colleagues have suggested that features of stimuli are bound into object files but that under certain circumstances feature codes may “migrate” to (i.e., are falsely considered as part of) other files that represent objects that actually do not carry the coded feature. Assuming that social events, like other people, are represented by event files, just like non-social events and objects (Hommel, 2018), Ma and colleagues hypothesized that feature codes bound to one event file may “migrate” to (i.e., are falsely treated as an ingredient of) another event file if the two files overlap in terms of their features – so that they are concurrently active. Using virtual reality, the authors presented human participants with a virtual face that moved either in synchrony with the participant or out of synchrony, assuming that synchrony creates a shared feature that facilitates feature migration between the representation of the virtual face and the self-representation of the participant. When the virtual face began to smile, participants reported better mood (an explicit measure of affect) and performed better in a brainstorming task (an implicit measure of affect), but only after having moved in synchrony with the virtual face. Along the same lines, Ma, Sellaro, and Hommel (2019) had a virtual face move in synchrony with the human participant or out of synchrony before morphing it into an ape’s face. In a subsequent intelligence test, participants scored significantly lower after having moved in synchrony with the virtual face. These studies suggest that sharing movement features promotes the migration of features like happiness or intelligence from the representation of another (virtual) individual to the representation of oneself.

Applying this rationale to the topic of attitudes suggests that one’s attitude towards an individual or an object might be affected by features that actually belong to the representation of another event, if the two representations share features. That judging people and things can be affected by events they are associated with has frequently been observed, such as with examples of the so-called Halo effect – which refers to the finding that positive (or negative) judgments of some characteristics of a person are overly likely to come with positive (or negative) judgments of other characteristics of this person (Thorndike, 1920; Nisbett & DeCamp Wilson, 1977). However, accounts of such effects have merely characterized them as exemplars of more general (equally unexplained) psychological biases (Feeley, 2002) rather than providing a transparent mechanism generating them. Therefore, we were interested to see whether our mechanistic account would allow us to generate particular biases experimentally – through feature migration.

**Fig. 1:**
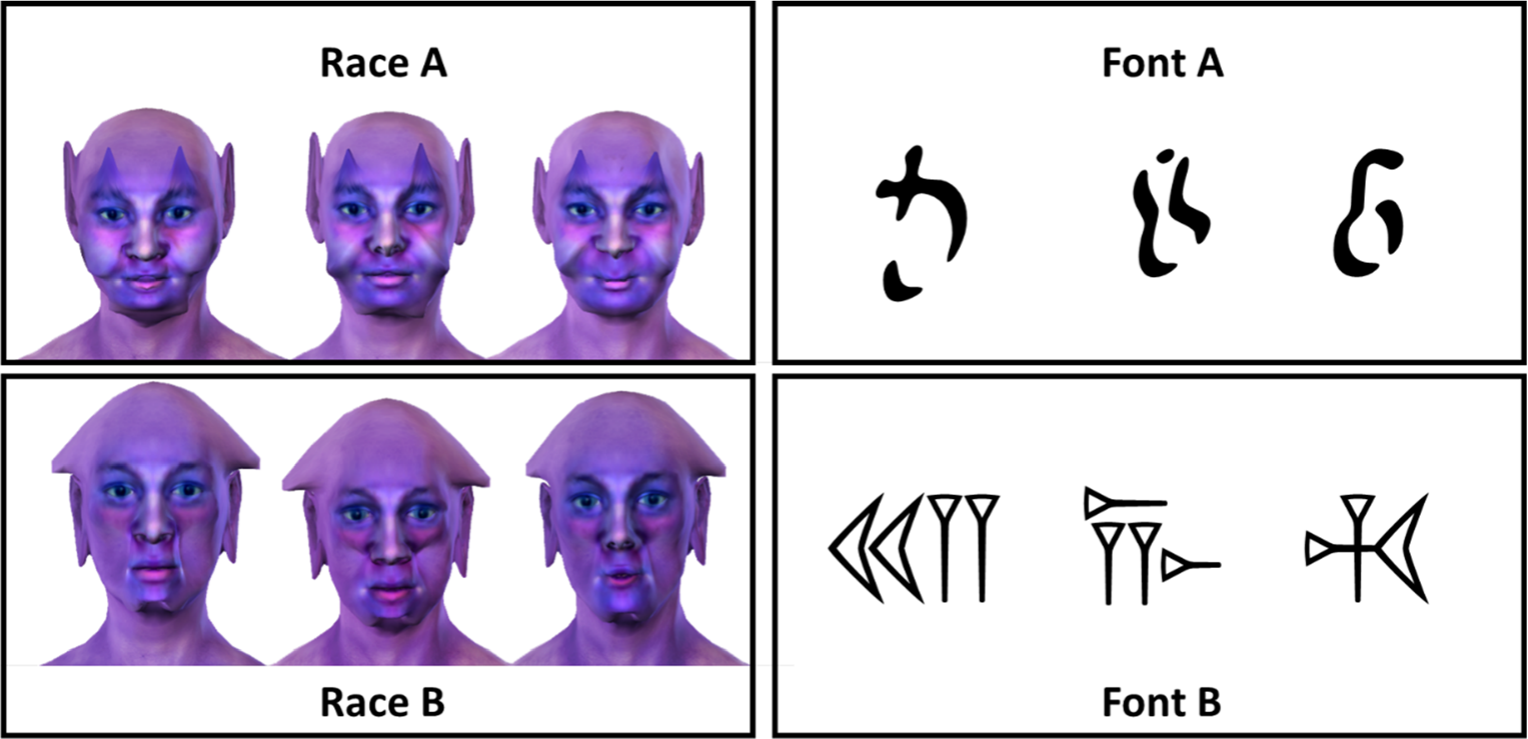
Three exemplars from each of the two fictitious races used in Experiment 1 and the two fonts used in Experiment 2

In Experiment 1, we confronted participants with exemplars of two fictitious “races.” Relations to human ethnicity were deliberately avoided, so to prevent the activation of specific racial stereotypes and the ethically problematic transfer of experimentally induced effects to real life. Accordingly, we created physically distinguishable “tribes” of individuals that were similar to outer-space characters in science fiction movies (see Fig. 1). While it seems impossible to create truly neutral and yet somewhat human-like characters, we tried to construct the members of the two races in such a way that they would not differ strongly in their likability.

In the first phase of the experiment (the acquisition phase), participants categorized the members of the two races by pressing a left or right key, respectively. We also presented participants with pictures of the International Affective Picture System (IAPS) that were chosen for their substantial positive or negative valence, and had participants categorize these pictures through reporting their valence by pressing a left or right key, respectively. Performing these tasks should, theoretically speaking, induce the creation of event files that would bind the perceptual features of the members of the two races and of the positive and negative IAPS pictures to the action they were categorized with – that is, to left and right keypresses. The event files would also include affective features. These should be relatively neutral for the outer space characters but should differ substantially in valence for the IAPS pictures. The feature-migration hypothesis would predict that the positive and negative features bound to the event files of the IAPS pictures should be more likely to migrate to the event files of those outer space characters that required the same keypress. Let us assume that race A and positive IAPS pictures would be categorized by pressing the left key. Accordingly, processing the features defining race A would tend to reactivate event files containing codes of these features, which in turn would contain codes representing the left-hand keypress. Activating these latter codes would tend to reactivate other event files containing the same codes, which in the example would be event files that all contain affective codes with positive valence. The feature-migration hypothesis implies that these active affective codes would function as if they would be part of the representation of the currently perceived member of race A (i.e., the participant would take this code to represent the present stimulus), so that the participant would rate this member as more likable than if this affective code would not have been activated.

To assess these predicted effects of feature migration, we presented participants again, after a short break, with the pictures presented in the first phase, and had them rate the pictures in terms of likability on a Likert-type scale (the test phase). For the IAPS pictures, this amounted to a mere manipulation check, as we obviously expected positive pictures to be liked better than negative pictures. More interesting were the ratings of the members of the two races. Notwithstanding possible main effects reflecting overall differences in likability of one race or the other, our feature-migration hypothesis predicts that members of the race that was categorized by pressing the same key as for positive IAPS pictures would be liked better than members of the race that shared the key with the negative IAPS pictures.

The reader might have noticed that the basic structure of the acquisition phase corresponds to what is known as the Implicit Association Test (IAT; Greenwald, McGhee, & Schwartz, 1998). As in our setup, this test also requires participants to respond to positive and negative material and to stimuli related to possible stereotypes, like racial prejudice, by means of the same response keys. The common observation is that performance is better if participants respond to their relevant ingroup (e.g., light-skinned individuals) by means of the same key as to positive material and to the relevant outgroup (e.g., dark-skinned individuals) by means of the same key as to negative material, as compared to a condition in which the mapping is reversed. The relative performance benefit in the former as compared to the latter condition is considered to represent the amount of pro-ingroup/anti-outgroup bias. The similarity between this design and the one we used in our study notwithstanding, it is important to emphasize that we did not use this design to *measure a pre-existing bias*, which is the ambition of the IAT, but to *induce a novel bias* in an experimentally controlled fashion.

## Experiment 1

### Method

#### Participants

Given the absence of similar previous studies, which made it hard to predict effect sizes and power, we decided to test about 200 (valid) participants per experiment. In total, 223 volunteers (149 male, age: M = 31.406 years, SD = 9.20 years) completed the first experiment; 200 passed the >75% accuracy criterion in the acquisition phase.

#### Materials

We created two stimulus sets: one for the positive-negative judgment and one for the two-races judgment. The positive-negative set was taken from the IAPS collection (Lang, Bradley, & Cuthbert, 1999). We removed similar, easy-to-confuse pictures and pictures showing faces of humans and other mammals, had three raters exclude pictures that were potentially disturbing, and then selected the 20 pictures with the highest and the 20 pictures with the lowest valence ratings. The two-races set was generated by creating two visual prototypes by using the software CrazyTalk8 and then creating 20 exemplars from each of the prototypes by systematically varying facial features.

Stimuli appeared at the center of the screen and “E” and “I” keys of the computer keyboard were used as left and right response keys, respectively. Participants were reminded of the stimulus-key mapping by two continuous displays at the top left and top right of the screen, showing the messages “press E for POSITIVE and <prototype picture of one race>” and “press I for NEGATIVE and < prototype picture of other race>.” The original resolution of the stimuli was 400 × 400 pixels, but we scaled the images depending on the resolution of the different monitors used by the participants by asking them to hold their credit/debit card against the screen and adjusting the size of a rectangle so that it would match the size of the card.

#### Procedure

The experiment was carried out using the online crowdsourcing service Amazon Mechanical Turk (MTurk). Participants filled in the informed consent form and were then instructed that this experiment would consist of two parts, and that the first part would require them to categorize two sets of stimuli by using a left and a right key. The stimulus sets were explained by means of four example pictures, two showing a positive and a negative object/event and the other two showing exemplars of the two fictitious races.

Then participants practiced categorizing the positive and negative pictures in one ten-trial block and categorizing the exemplars of the two fictitious races in another ten-trial block. The order was balanced across participants. Next, the two tasks were combined and practiced in another 16-trial block. In each trial, the stimulus was presented until response and the inter-trial interval was 250 ms. This was also the case in the experimental 80-trial acquisition block (40 with pictures from the positive-negative set and 40 with pictures from the two-races set) that followed. The order of stimuli was random except that there were no more than two repetitions of the same stimulus set (or judgment). The mapping of the positive and negative judgments to the two keys was counterbalanced across participants, as was the combination of the keys used for positive and negative judgments (“positive” and “negative” keys for short) and the keys used for the two races – i.e., across participants, each race was assigned to the “positive” and the “negative” key with about equal likelihood (105 participants made race-A responses with the “positive” key and 95 made race-B responses with the “positive” key).

After the acquisition phase was completed, participants watched a 10-min video on the chemistry of color of YouTube channel “artregeous with Nate” (used with permission from the creator). After watching the video, the participants were asked two questions regarding the content, to check whether they had paid attention. The purpose of this video was to reduce possible transfer effects from the acquisition to the test phase. In the test phase, participants were instructed to rate the 40 pictures showing exemplars of the two fictitious races and the 40 IAPS pictures they had just encountered in the acquisition phase by using the keyboard’s number keys (1–7) to respond to a 7-point Likert-type scale (ranging from “dislike very much” to “like very much”). Again, the order of stimuli was random except that there were no more than two repetitions of the same stimulus set. Stimuli remained on-screen until a response was made. Participants were then thanked and debriefed. The entire session took about 15–20 min.

## Results

Data from all participants who passed our accuracy criterion were analyzed. In the acquisition phase, categorization accuracy was high for all four categories: positive pictures (94%), negative pictures (93%), race A (see top row of Fig. 1: 93%), and race B (see bottom row of Fig. 1: 93%). While the exemplars of the two fictitious races were constructed to look as emotionally neutral as possible, facial configurations may always be perceived as more positive or negative. To test for this possibility, we ran ANOVAs on the four accuracy scores as a function of key combination (race A vs. B categorized with the “positive” key). Results revealed a significant effect of key combination for accuracy on race A, F(1,198) = 5.47, p = .02, indicating that accuracy on this race was somewhat higher when it was categorized with the same key as positive events (94%) than when it was categorized with the same key as negative events (92%).

**Fig. 2:**
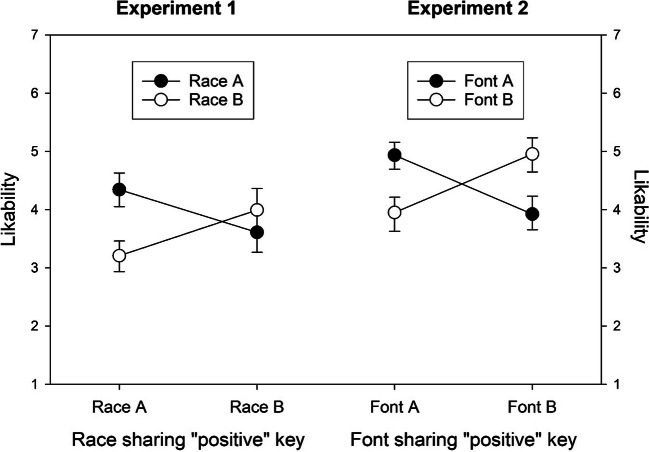
Likability ratings (with 95% confidence intervals) of members of the two fictitious races used in Experiment 1 and the two fonts used in Experiment 2

The ratings from the test phase underwent ANOVAs with valence or race as within-participant factor and key combination (race A vs. B categorized with the “positive” key) as between-participant factor. The race-B ratings from one participant were missing, so that this participant was not considered in the race-related analyses. The analysis of the ratings of the positive-negative IAPS pictures yielded the expected, highly significant main effect of valence, F(1,198) = 897.53, p < .001, confirming that positive pictures were liked better than negative pictures (5.9 vs. 2.4), while the main effect of key mapping and the interaction were far from significant, ps > .2. More interestingly, the analysis of the ratings of the members of the two fictitious races yielded a highly significant main effect of race, F(1,197) = 11.05, p = .001, and a highly significant interaction, F(1,197) = 57.27, p < .001. As shown in Fig. 2, there was a general preference for members of race A and a specific preference for members of the race that was paired with the “positive” key. Separate two-tailed t-tests confirmed that the key-mapping effect was highly significant for both races, t(198) = 3.45, p = .001, t(197) = 3.54, p < .001.

## Discussion

We assumed that introducing a feature that is shared between pictures of positive affective valence and members of one race, and between pictures of negative affective valence and members of the other race, should promote feature migration between event files including shared features. As a consequence, members of the race sharing a key with positive pictures should receive higher likability scores in the test phase than members of the race sharing a key with negative pictures. This prediction was clearly confirmed. In Experiment 2, we tried to generalize this finding to non-social material by replacing the two fictitious races by two unfamiliar fonts.

## Experiment 2

### Method

In total, 240 volunteers (146 male, age: M = 37.45 years, SD = 9.04 years) completed the study; 204 passed the >75% accuracy criterion in the acquisition phase (105 with font A mapped to the “positive” key). The method was exactly as in Experiment 1, except that the pictures of the fictitious races were replaced by meaningless symbols, which we took from the cuneiform script (en.wikipedia.org/wiki/Cuneiform) and the imaginary script wizard speak (www.1001freefonts.com/de/wizard-speak.font).

## Results

Data were analyzed as in Experiment 1. In the acquisition phase, categorization accuracy was high for all four categories: positive pictures (93%), negative pictures (93%), font A (see top row of Fig. 1: 97%), and font B (see bottom row of Fig. 1: 97%). ANOVAs on the four accuracy scores as a function of key combination (font A vs. B categorized with the “positive” key) revealed no significant effect of key combination, ps > .29.

The ratings from the test phase underwent ANOVAs with valence or font as within-participant factor and key combination (font A vs. B categorized with the “positive” key) as between-participant factor. The analysis of the ratings of the positive-negative IAPS pictures yielded the expected, highly significant main effect of valence, F(1,202) = 819.85, p < .001, confirming that positive pictures were liked better than negative pictures (6.0 vs. 2.5), while the main effect of key mapping and the interaction missed significance, ps > .06. More interestingly, the analysis of the ratings of the symbols yielded a highly significant interaction effect, F(1,202) = 55.43, p < .001, while the main effects were not significant, ps > .85. As shown in Fig. 2, there was a specific preference for symbols from the font that was paired with the “positive” key. Separate two-tailed t-tests confirmed that the key-mapping effect was highly significant for both fonts, t(202) = 5.46, p < .001, t(202) = 4.86, p < .001.

## Conclusions

The purpose of this study was to see whether consistent judgment biases can be experimentally induced through feature migration (Ma et al., 2016, 2019). Both experiments show that sharing a superficial feature with other, entirely unrelated, events is sufficient to bias the likability of (pictures of) members of a fictitious race and symbols of an unfamiliar or fictitious font. Our findings as such are not novel at all: we do know that people like and dislike others and there can be little doubt that implicit and/or explicit learning processes are responsible for that. The novelty we rather see in the fact that mechanistically explicit cognitive theorizing that originally was developed to account for the processing of simple visual features (like Treisman’s feature migration account) can account for a central concept of social-psychological thinking, like attitudes. From that perspective, we would like to point out five further implications.

First, the induced biases were very similar in type and degree, except for the slight overall preference for members of race A in Experiment 1. This suggests that the assumed migration effect is similar for representations of social and non-social events, as claimed by Hommel (2018). It is important to emphasize that there is still a gap between the pictures of fictitious characters used in this study and between real, living individuals. While closing this gap will be a necessary next step, there are obvious ethical limitations if it comes to the induction of biases pro or against living beings. Hence, tackling this empirical challenge will require considerable ethical sensitivity and experimental sophistication. Nevertheless, the present findings support the idea that non-social cognitive theory can account for the mechanisms underlying social behavior (Kim & Hommel, 2019). We believe that using a mechanistically elaborate and transparent theory to account for social phenomena stimulates theorizing and exploiting experimental paradigms across domains and sub-disciplinary borders. In that sense, we see our approach fully in line with the original, but somewhat forgotten, theoretical and experimental ambitions of the original social-cognition approach of the 1980s.

Second, it is important to emphasize that our predictions were not derived from a theory dedicated to understanding attitudes, opinions, and preferences. Rather, these predictions emerged as a byproduct of a theory that originally aimed to account for the interplay between perception and action in rather simple tasks (Hommel et al., 2001). This suggests that piecemeal theorizing is unnecessary to account for social behavior, as is the introduction of novel theoretical assumptions or concepts, which in turn encourages attempts to parsimoniously account for other, apparently related phenomena by means of the same theoretical toolbox. An obvious target would be the Halo effect, as mentioned already, and tackling prejudice or stereotypes seems feasible.

Third, while we are optimistic that our study targeted the actual mechanisms underlying attitudes and related preferences, it goes without saying that our use of artificial, rather meaningless stimulus material is likely to have only scratched the surface of everyday discrimination of minorities and other attitude-related behaviors. Real-life attitudes are acquired over a much longer time and unlikely to be as isolated as the preference towards particular outer-space figures. Rather, they will be integrated into more complex worldviews and value systems that are likely to be more resistant to change than the associations created in our study.

Fourth, and relatedly, our study provides evidence for experimentally induced biases that were robust enough to survive a couple of minutes. For many researchers, the concept of attitude implies a substantially longer time range than that, which calls for further studies testing whether feature migration can also be demonstrated if acquisition and tests are separated by hours, days, and months.

Finally, given the strong similarities between the setup of our acquisition phase and the IAT, it might be considered worrying that we managed to actually *induce* affective biases in a task that is commonly used to *measure* these biases. On the one hand, our study was not designed to assess this connection to the IAT, and our findings do not rule out the possibility that pre-existing biases might still show effects over and beyond the effects of our induced biases. On the other hand, however, our findings do raise the possibility that the IAT might induce a kind of Heisenberg phenomenon – the possibility that the measurement process affects what is being measured.
